# Investigation of running foot strike technique on Achilles tendon force using ultrasound techniques and a Hill-type model

**DOI:** 10.1186/1757-1146-5-S1-P25

**Published:** 2012-04-10

**Authors:** Sarah M Stearne, Jonas Rubenson, Jacqueline Alderson

**Affiliations:** 1 The School of Sport Science Exercise and Health, The University of Western Australia, Perth, WA, 6009, Australia

## Background

It is reported that 75% of long distance runners use a rearfoot strike (RFS) technique. This percentage decreases in faster runners, where the incidence of midfoot and forefoot strikers (FFS) increases [1]. It is possible that FFS better utilises the passive-elastic mechanisms of the lower limb reducing energy cost. Williams *et al. *[2] found runners who converted from RFS to FFS during a single training experienced increased fatigue and delayed onset muscle soreness in the calf musculature, indicating increased muscle work. This research aims to investigate the role of the Achilles tendon and triceps surae muscles in FFS versus RFS running hypothesising that the FFS will have increased Achilles tendon force.

## Materials and methods

Natural FFS (n=9) and RFS (n=9) distance runners ran on a treadmill at 3ms^-1^ while muscle fibre length change of the medial and lateral gastrocnemius and soleus were recorded using ultrasound and muscle activation via surface electromyography. Individual contribution of the triceps surae muscles to Achilles tendon force was determined using a Hill-type model based on muscle activation, the muscles force-length-velocity relationship (from an individually scaled musculoskeletal OpenSim model), maximum isometric muscle force and pennation angle. Achilles tendon and triceps surae individual muscle forces were recorded while runners performed their natural (fore or rearfoot) and then converted to their unnatural strike.

## Results

**Figure 1:**
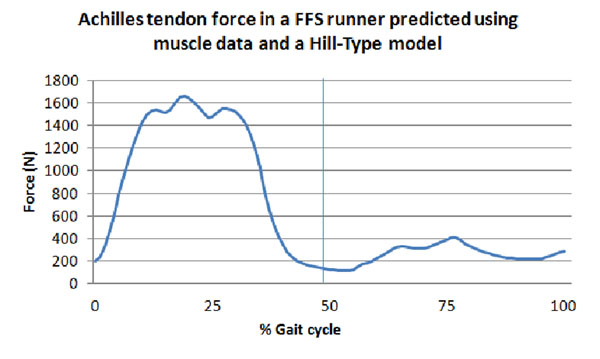


**Figure 2:**
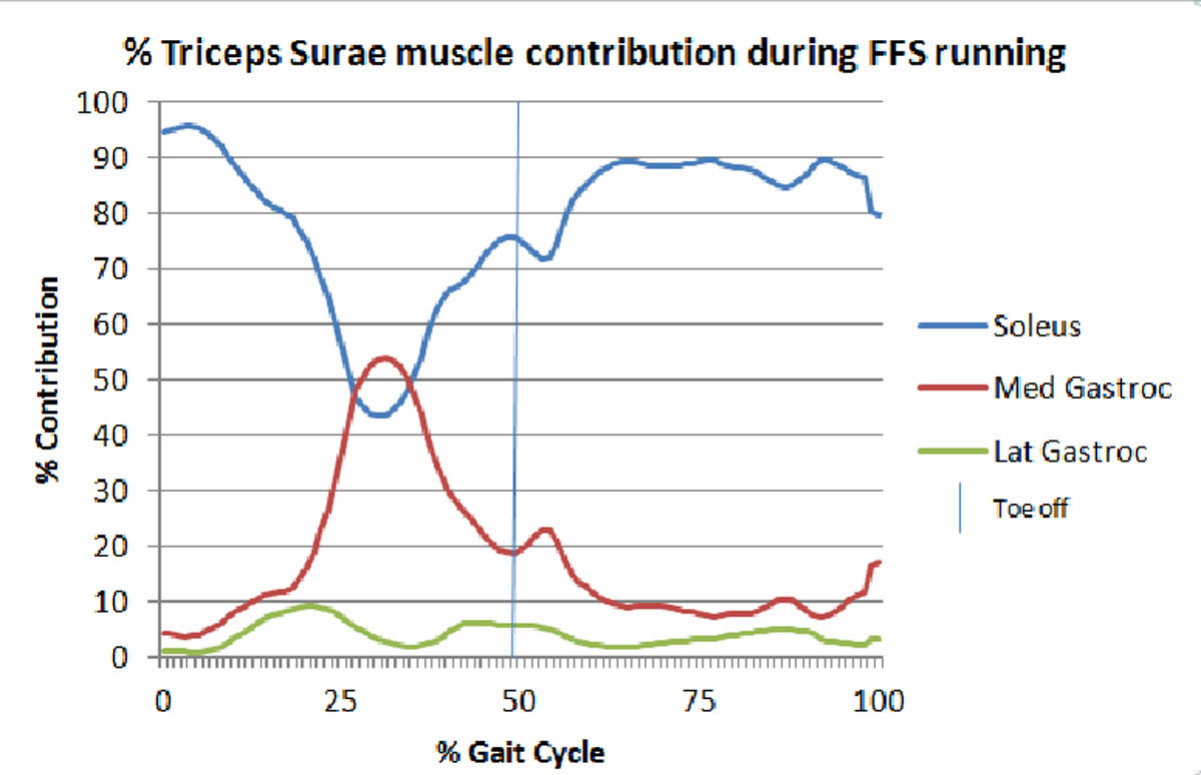


Results from one FFS participant are presented below (Figure 1 &2), additional data is being processed. Preliminary results indicate tendon force is lower than RFS results in the literature.

## Conclusions

This research provides insight into the role of the Achilles tendon during FFS running and sheds light on its’ contribution to reducing energy cost. It also reveals the altered demand on the triceps surae muscles which may have implications for technique recommendations and training requirements.
